# Preparation and execution of teeth clenching and foot muscle contraction influence on corticospinal hand-muscle excitability

**DOI:** 10.1038/srep41249

**Published:** 2017-01-24

**Authors:** Naeem Komeilipoor, Risto J. Ilmoniemi, Kaisa Tiippana, Martti Vainio, Mikko Tiainen, Lari Vainio

**Affiliations:** 1 Division of Cognitive and Neuropsychology, Institute of Behavioural Sciences, University of Helsinki, Finland; 2 Department of Neuroscience and Biomedical Engineering, Aalto University School of Science, Espoo, Finland; 3 Phonetics and Speech Synthesis Research Group, Institute of Behavioural Sciences, University of Helsinki, Finland; 4 Department of Modern Languages, University of Helsinki, Finland

## Abstract

Contraction of a muscle modulates not only the corticospinal excitability (CSE) of the contracting muscle but also that of different muscles. We investigated to what extent the CSE of a hand muscle is modulated during preparation and execution of teeth clenching and ipsilateral foot dorsiflexion either separately or in combination. Hand-muscle CSE was estimated based on motor evoked potentials (MEPs) elicited by transcranial magnetic stimulation (TMS) and recorded from the first dorsal interosseous (FDI) muscle. We found higher excitability during both preparation and execution of all the motor tasks than during mere observation of a fixation cross. As expected, the excitability was greater during the execution phase than the preparation one. Furthermore, both execution and preparation of combined motor tasks led to higher excitability than individual tasks. These results extend our current understanding of the neural interactions underlying simultaneous contraction of muscles in different body parts.

It is well established that contraction of a muscle modulates the corticospinal excitability (CSE) not only of the contracting muscle^1^,^2^, but also of the resting muscles located in remote parts of the body; this is the so-called “*remote effect*”^3^,^4^,^5^,^6^. The effect of muscle contraction on CSE can be investigated by examining the size of the motor evoked potential (MEP) elicited by transcranial magnetic stimulation (TMS) over the primary motor cortex (M1). The MEP reflects the net effect of excitatory and inhibitory inputs on the descending corticospinal pathway^7^. MEPs evoked in actively contracting muscles are larger and earlier than that in resting muscles due to higher levels of activity in their motor neuron pools^8^. Intriguingly, increased MEPs have been observed in arm and hand muscles during teeth clenching^3^ or vocalizing^9^, and also during contraction of the eye^4^, foot^5^, opposite limb^10^ or elbow^11^. Furthermore, MEPs in leg muscles are facilitated by handgrips^6^ and teeth clenching^3^. Moreover, unilateral muscle contraction facilitates the MEP in the muscles of both ipsilateral and contralateral sides of remote segments^12^,^13^. It has also been shown that the magnitude of MEP facilitation varies as a function of the strength of voluntary muscle contraction—the stronger the muscle contraction is, the larger the amplitude of the MEP would be^13^,^14^. Taken together, the aforementioned studies suggest that motor functions of different body parts are not embedded in the brain as separated units but closely interact. However, the mechanisms of these interactions remain to be elucidated.

In particular, investigating the *remote effect* during preparation and execution of simultaneous tasks in different muscles would provide clues about the motor-level interrelationships among the neural mechanisms underlying simultaneous production of separate movements. Experiments with functional magnetic resonance imaging (f MRI) have shown that combined movements of the wrist and the foot result in greater activation in contralateral M1, compared to the activations found with single movements^15^. Similarly, in a positron emission tomography (PET) study, simultaneous, versus isolated, movements of wrist and ankle led to stronger activities in M1^16^. Moreover, in a recent TMS study, ipsilateral facilitation of wrist representations in M1 increased significantly when participants performed difficult hand–foot coordination tasks as compared to simple hand movements^17^. Taken together, these findings suggest that M1 plays an important role in mediating the coordination of simultaneous, versus isolated, movements. However, it is not clear whether movements of body parts alone, and in combination, would result in different patterns of MEP modulations in the non-homologous remote segments.

The generation of voluntary movements starts with preparatory activities in motor areas such as primary and premotor cortex^18^. Preparatory changes in M1 before the execution of a movement have been documented in both humans^19^,^20^ and monkeys^18^,^21^. It has been suggested that these preparatory activities might arise concurrently in multiple motor areas^18^, irrespective of their ‘remoteness’ from the muscles to be moved^21^. In addition, TMS studies have shown that before movement execution, MEPs are facilitated in active^22^ and agonist muscles^23^,^24^, whereas they are inhibited in antagonist^24^,^25^ and contralateral homologous muscles^23^. However, it is not clear whether movement preparation would lead to modulation of CSE in resting muscles located in non-homologous remote segments, and whether preparation of simultaneous, versus isolated, movements would result in different patterns of CSE in these resting muscles.

Regardless of the exact mechanism that lies behind the *remote effect,* this phenomenon can have practical implications in clinical settings. For instance, it has been proposed that motor training of an unaffected (or less affected) limb might be advantageous for improving motor recovery of a limb after a stroke or spinal cord injuries^26^. Moreover, boosting corticospinal excitability through contraction of muscles in remote parts of the body could be beneficial since in patients with central nervous system diseases, it may be challenging or even impossible to elicit reproducible MEPs in target muscles^4^. Additionally, facilitation fortified by contraction of remote muscles than target ones may be beneficial since muscle activation does not interfere with the baseline of the signal. Hence, the potential finding of the CSE of a resting muscle reinforced by combined action of two remote body parts may have clinical implications.

The aim of the present study was to investigate to what extent the CSE of a hand muscle is modulated during preparation and execution of simultaneous movements of non-homologous body parts. To do so, we chose foot dorsiflexion and teeth clenching as motor acts and FDI as a hand muscle since it has already been shown that MEPs in FDI are facilitated by foot dorsiflexion (e.g., ref. 27) and teeth clenching alone (e.g., ref. 3). We investigated the extent to which the CSE of a resting hand muscle during preparation and execution of teeth clenching and foot dorsiflexion (alone or in combination) is different from that during the baseline condition of merely observing a fixation cross. We expected MEPs to increase less during the preparation rather than during motor-task execution; in both cases, we also expected them to be larger than those recorded during fixation-cross observation. Furthermore, we expected that combined motor acts would lead to higher excitability than individual acts in isolation.

## Results

**Figure 1:**
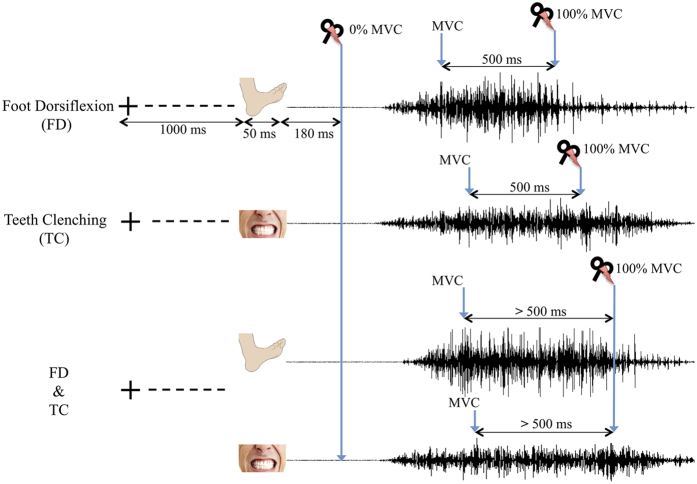
Scheme of the experimental protocol during experimental blocks. The two-headed horizontal arrows represent the timing of fixation cross and visual stimulus appearing on the monitor and TMS pulse delivery. A detailed description of the design is provided in the Materials and methods section.

We assessed changes in CSE of a hand muscle induced by preparation and execution of ipsilateral foot dorsiflexion (*FD*) and teeth clenching (*TC*) either separately or in combination (*FD & TC*). CSE was estimated based on MEPs elicited by TMS and recorded from the FDI muscle. The participants were asked to execute maximum voluntary contraction (MVC) of the three actions after the presentation of a visual stimulus depicting one of them. TMS pulses were delivered 180 ms after stimulus presentation (0% MVC) and after a sustained 500-ms MVC (100% MVC). Baseline MEPs were recorded during observation of a fixation cross (*Fixation*). See Fig. 1 for an overview of the protocol. It should be noted that positive and negative MEP values do not imply excitatory and inhibitory effects and indicate that the raw MEP amplitudes lie above and below the mean respectively.

**Figure 2:**
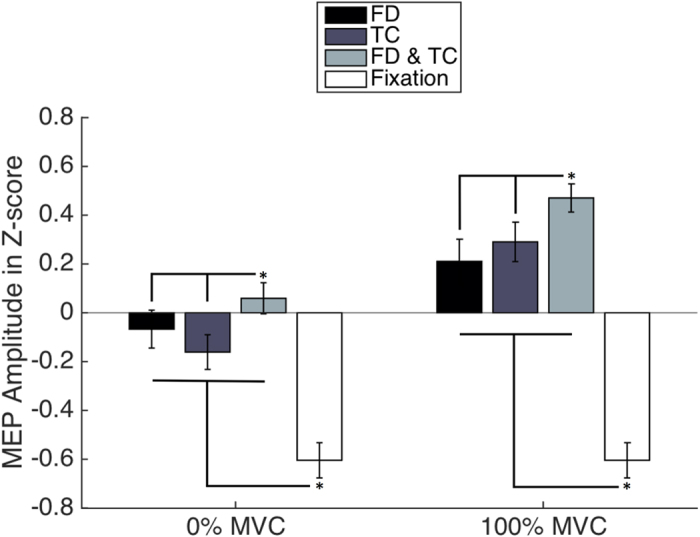
Grand-averaged (z-score of the) MEP amplitudes of FDI during observation of a fixation cross (*Fixation*), preparation (0% MVC) and execution (100% MVC) of right-foot dorsiflexion (*FD*), teeth clenching (*TC*) and simultaneous right-foot dorsiflexion and teeth clenching (*FD & TC*). During both 0% and 100% MVC, the excitability of the FDI muscle increased for *FD, TC* and *FD & TC* from the level during observation of the fixation cross (Fixation). Furthermore, execution and preparation of simultaneous movements (*FD & TC*) resulted in higher excitability than *FD* as well as *TC*. The error bars represent standard errors. *p < 0.05.

First, to test whether TMS stimulation and/or the tiredness resulting from several muscle contraction attempts altered corticospinal excitability, the MEP amplitudes recorded in the *pre*- and *post*-baseline blocks were compared by using a paired sample t-test. The analysis yielded no significant effect: motor excitability of the FDI muscle did not change from the *pre* (−0.52 ± 0.1) to the *post* block (−0.68 ± 0.08) [*t*_(16)_ = 1.24, *p* = 0.23, Cohen’s *d* = 0.62]. Next, we merged the two baseline blocks (now named *Fixation*) and performed two one-way repeated-measures ANOVAs to evaluate the differences in MEP amplitudes (in z-scores) recorded during 0% and 100% MVC conditions (*TC, FD* and *FD & TC*) and also those evoked during *Fixation*. It was found out that all the variables were normally distributed, as assessed by the Shapiro–Wilk test (*p* > 0.05), and that the assumption of sphericity had not been violated (*p* > 0.05). The one-way repeated-measures ANOVA for 100% MVC revealed that the effect of *condition* was significant (*F*_(1,16)_ = 28.97, *p* < 0.001, η^2^ = 0.64). Moreover, the observation of the *fixation* cross led to significantly smaller MEPs (*Fixation* = −0.6 ± 0.07) than simultaneous teeth clenching and right-foot dorsiflexion (*FD & TC* *=* 0.47 ± 0.06) [*t*_(16)_ = 9.74, *p* < 0.001, Cohen’s *d* = 4.87], teeth clenching (*TC* = 0.29 ± 0.08) [*t*_(16)_ = 8.9, *p* < 0.001, Cohen’s *d* = 4.45] alone or right-foot dorsiflexion (*FD* = 0.21 ± 0.09) [*t*_(16)_ = 5.53, *p* < 0.001, Cohen’s *d* = 2.76] alone. *FD & TC* together resulted in significantly larger MEPs than *TC* [*t*_(16)_ = 2.25, *p* = 0.039, Cohen’s *d* = 1.12 ] and *FD* [*t*_(16)_ = 2.85, *p* = 0.012] alone. There were no statistically significant differences between *TC* and *FD* [*t*_(16)_ = 0.6, *p* = 0.52, Cohen’s *d* = 0.3]. Also, for the 0% MVC the effect of *condition* was significant (*F*_(1,16)_ = 10.85, *p* = 0.005, η^2^ = 0.4). The observation of the *fixation* cross led to significantly weaker MEPs (*Fixation* = −0.6 ± 0.07) than preparation for the combined movement (*FD & TC* = 0.06 ± 0.06) [*t*_(16)_ = 5.73, *p* < 0.001, Cohen’s *d* = 2.86], teeth clenching (*TC* = *−*0.17 ± 0.07) [*t*_(16)_ = 3.3, *p* = 0.004, Cohen’s *d* = 1.65] or right-foot dorsiflexion (*FD* = −0.07 ± 0.08) [*t*_(16)_ = 4.3, *p* = 0.001, Cohen’s *d* = 2.15]. Preparation for *FD & TC* resulted in significantly larger MEPs than preparation for *TC* [*t*_(16)_ = 3.98, *p* = 0.001] and *FD* [*t*_(16)_ = 2.26, *p* = 0.04, Cohen’s *d* = 1.13] alone. There were no statistically significant differences between preparation for *TC* and *FD* [*t*_(16)_ = 1.6, *p* = 0.13, Cohen’s *d* = 0.8]. Thus, (1) the execution and preparation of all motor tasks resulted in higher excitability of the resting hand muscle than the observation of the fixation cross; (2) both the execution and preparation of combined tasks led to higher excitability than individual tasks in isolation (Fig. 2).

Finally, we performed a two-way repeated measures ANOVA with independent factors *movement (FD, TC and FD & TC*) and *force level* (0% and 100% MVC). It was found out that all the variables were normally distributed, as assessed by the Shapiro–Wilk test (*p* > 0.05), and that the assumption of sphericity had not been violated (*p* > 0.05). The main effects of *movement (F*_(1,16)_ = 11.76, *p* = 0.003, η^2^ = 0.424) and *force level (F*_(1,16)_ = 21.04, *p* < 0.001, η^2^ = 0.568) were significant. Hand cortical excitability was enhanced during the *FD & TC* as compared to *TC* [*t*_(16)_ = 4.36, *p* = 0.001] and *FD* [*t*_(16)_ = 3.4, *p* = 0.003, Cohen’s *d* = 1.7]. Moreover, the excitability was higher during the execution (100% MVC) than during preparation (0% MVC) of movements [*t*_(16)_ = 4.59, *p* < 0.001, Cohen’s *d* = 2.29]. The interaction between the *movement (FD, TC and FD & TC*) and *force level* (0% and 100% MVC) was not statistically significant (*F*_(1,16)_ = 1.63, *p* = 0.22, η^2^ = 0.093).

## Discussion

We used TMS to investigate how the preparation and execution of teeth clenching and right-foot dorsiflexion alone or in combination influence CSE of left hand M1. We found larger facilitation of CSE during execution and, to a lesser extent, preparation of all the motor tasks than observation of a fixation cross. Furthermore, the execution and preparation of combined motor tasks resulted in greater increase of CSE than individual tasks in isolation. These findings may be relevant to ongoing efforts to understand the mechanism of the *remote effect* underlying preparation and execution of simultaneous movements of different body parts.

Different studies have indicated that the contraction of a muscle modulates the CSE of M1 representations of resting muscles in remote segments of the motor cortex^3^,^4^,^5^,^6^. Our results expand on these findings by showing that (1) the *remote effect* occurs even during the preparation of the movement and (2) both the preparation and execution of simultaneous motor tasks of different body parts impose an additional effect on the excitability of M1 representations of resting muscles, as compared to motor tasks performed in isolation. Previous studies have suggested that a functional neuronal network between supplementary motor area (SMA), premotor cortex (PMC) and M1 might be responsible for the processing of simultaneous movements of different effectors^15^,^16^. To what degree this network is responsible for the enhancement of CSE in resting hand M1 during simultaneous movements of foot and teeth clenching should be addressed in future studies using functional neuroimaging methods such as fMRI or EEG in combination with repetitive TMS (rTMS).

The amplitude of MEPs induced by TMS provides a measure of corticospinal excitability, which is influenced by the excitability of neurons both in the motor cortex (cortico) and in the motor-neuron pool (spinal)^28^. Hence, the *remote effect* might result from both cortical and subcortical facilitation mechanisms. Cortical influences are suggested to be related to non-selective activation enforced by the supplementary motor area (SMA)^4^, neuronal network between dorsal premotor cortex (PMd), SMA and M1^27^ or input from neighboring areas within M1^3^. Using paired-pulse TMS, Byblow *et al*.^27^ showed that a conditioning pulse over PMd and SMA modulates the excitability of the resting hand M1 area^27^, suggesting that these areas indeed belong to the network responsible for this effect. Subcortical facilitation mechanisms also have been suggested to contribute to the non-specific facilitation of muscle contraction. It has been shown that F-waves, used as an index of motor-neuron-pool excitability, increase in the resting arm and hand muscles during teeth clenching^3^ and voluntary contraction of the ipsilateral arm^29^. Overall, these results imply that the *remote effect* results from both cortical and subcortical mechanisms; however, the relative contribution of each mechanism is not known (for a review, see ref. 26).

Based on the results of the present study, we cannot elucidate the exact neuronal mechanism underlying the *remote effect,* such as interaction among representation of muscles belonging to different body parts, since we just stimulated the left hand M1 area during the right foot dorsiflexion and teeth clenching and recorded the MEPs in the right hand. The recorded MEPs might have been influenced by the interaction between M1 and other cortical and subcortical regions. It has been suggested that the output of M1 results from a net effect of several specific interactions including intrahemispheric (within M1), interhemispheric (M1 to M1) and interregional (e.g., premotor cortex or cerebellum to M1) interactions^30^. Speculating about details of these interactions during the *remote effect* is beyond the scope of the current study, not least because TMS MEP recordings have certain limitations. Future studies should address the mechanism of the *remote effect* with high spatiotemporal resolution, but we suggest employing an experimental protocol that includes movements of body parts alone and in combination as it allows for tackling the integration of inputs from neighboring areas within the motor cortex.

A key finding in the present study is that the *remote effect* of muscle contraction exists, albeit to a lesser degree, even during the movement preparation phase: hand MEPs during preparation of simultaneous and isolated teeth clenching and ipsilateral foot dorsiflexion were larger than those recorded during the fixation-cross condition. The preparatory modulation of CSE has already been shown in active muscles^22^,^23^,^31^, agonist/antagonist muscle pairs^23^,^25^, and contralateral homologous muscles^23^. Our results corroborate these findings and indicate that the preparation of simultaneous and isolated movements of different body parts lead to the facilitation of CSE in resting muscles located in non-homologous remote segments. Remarkably, similar to the results obtained during movement execution (100% MVC), preparation of combined movements (0% MVC) resulted in larger MEPs than the individual ones which produced comparable facilitation of CSE. This implies that preparation of simultaneous, versus individual, movements would lead to increased excitability in M1 representations of resting muscles.

The existence of these cross-talk effects during voluntary muscle contractions might suggest the presence of connections between motor representations^32^. Although the functional organization of M1 clearly induces separate representations of face, arm and leg^33^, representations of muscles in the same and different body parts overlap^34^,^35^. In monkeys, movements of different digits activate the same individual neurons in M1, and the areas activated during movements of different digits exhibit extensive overlap^36^. Also, fMRI in humans has revealed overlapping and distributed patterns of activity in M1 for movements of the fingers, wrist, and elbow^37^,^38^. Additionally, electric stimulation of some sites within M1, both in humans^39^ and monkeys^32^, produce synergetic movements such as a closing hand moving toward an opening mouth. Thus, there is a high degree of overlap in the representation of muscular groups, meaning that a distributed network in M1 controls different body segments, possibly in order to favor the production of multi-segment motor synergies^40^. The effect of muscle contraction on the facilitation of MEPs in remote segments when the test muscle is at rest suggests that the neuronal networks responsible for controlling movements of different body parts are not embedded in the brain as separate modules, and that they closely interact even in M1 representation of resting muscles. Whether the *remote effect* induces activation of appropriate muscles to support motor coordination functions such as multi-limb movement remains to be elucidated.

It should be noted that we presumed that subjects performed only teeth clenching in *TC*, only dorsiflexion in *FD*, and only teeth clenching and dorsiflexion in *FD &TC* tasks. However, this was not fully guaranteed because EMG was taken only from right FDI, MS, and TA. In general, isometric maximal voluntary contraction of muscles around one joint induces activities of muscles around other joints of the same limb as well as those around the target joint. For instance, it has been shown that maximum voluntary teeth clenching induces contraction of trunk muscles^41^. Hence, the execution of MVC possibly led to the activity of some other muscles along with TA and MS, which contributed to the CSE excitability found during the MVC. Moreover, it should be acknowledged that recording the rest condition during the experimental blocks (e.g., by delivering TMS pulses before visual stimulus) might have been a better choice to ensure that other factors such as the level of arousal did not affect the difference found in MEP amplitudes between rest and task conditions. We recorded two baseline (rest) conditions before (*pre*) and after (*post*) the last experimental blocks. The comparison of the MEP amplitudes of the *pre* and the *post* baselines yielded no significant effect, implying that the overall excitability of the corticospinal system remained unchanged over the course of the experiments, and that factors such as TMS stimulation and/or the tiredness resulted from several muscle contraction attempts did not affect the level of excitability in the resting hand muscle. Tazoe *et al*.^14^ have reported that voluntary isometric knee extension facilitated wrist flexor MEP with linear relationship to knee extension force, and it remained unchanged even when knee extension force decreased due to central and peripheral muscle fatigue. It has been proposed that lack of reduction in MEP facilitation during the presence of fatigue may imply that the M1 is the source of neural generation of MEP facilitation in a remote segment^26^. Moreover, Tazoe *et al*.^13^ recorded the resting/control trials randomly throughout a session; TMS was delivered at hand motor area, and MEPs were recorded from FDI muscle (at rest or active) while the ankle was at rest and compared it with those recorded during dorsiflexion of ankle. They found that in the control trial, there was no significant difference in the amplitudes of the MEPs recorded from FDI when the test muscles were at rest or active while they were significantly smaller than MEPs recorded during dorsiflexion of ankle^13^. Therefore, this implies that the increment of excitability in resting muscles during contraction of remote segments could not simply be due to the anticipation of the various conditions or arousal level.

However, arousal phenomena might have a general facilitatory effect during the *remote effect* since during the contraction participants go from a relaxed resting state into a state of preparedness and general attention. It has been shown that mental activity may lead to corticospinal excitability changes^42^. Rossini, *et al*.^42^ found that MEP amplitudes in forearm muscles were larger when subjects looked at a target and performed mental calculations, compared to a condition with eyes closed while trying to avoid mental activity. Moreover, facilitation of F waves during mental arithmetics has been reported^4^. Thus, arousal may have an influence on the modulation of excitability both at cortical and spinal levels. It has also been shown that the arousal level can modulate the amplitude of movement-related potentials during action preparation and late movement execution with no significant difference during the two phases^43^. On the other hand, higher level of arousal was reported during movement preparation than execution^44^. In the present study, however, the level of excitability was higher during execution than during preparation of the movement and in both cases it was larger than the level of excitability during fixation-cross observation. Hence, it is unlikely that the modulation of CSE found in the present study is merely due to the increase in the level of arousal. However, the larger MEPs during simultaneous movements might have been affected by the higher level of arousal during combined movements. It has been shown that pupil dilation, as a measure of arousal, increased in amplitude and latency as a function of movement complexity^45^. Further research, possibly by measuring other physiological responses such as movement-related potentials, skin conductance and pupil dilation along with MEPs, is needed to understand the intricate mechanisms through which arousal may modulate CSE excitability of a resting muscle during both simultaneous and isolated movements of different body parts.

Whether the non-specific facilitation found in the present study is influenced by intracortical or subcortical mechanisms or by the inputs from neighboring areas within the motor cortex itself or by the level of arousal or all of the above, it opens new research avenues. For instance, the higher CSE of a resting hand muscle induced by combined movements of feet and mouth may have resulted from the summation of activities that spread in the opposite direction from mouth and feet to hand regions. It has been demonstrated that neural activity initiated within a small motor cortical locus of cats spreads to neighboring regions in which a variety of muscles are represented^46^. It is plausible that similar neural mechanism of spread of activity within M1 exists in humans, too. One possible way to test it is to record electrical activities from the M1 using intracranial EEG during preparation and execution of simultaneous tasks in different muscles. Moreover, it has been shown that in the interactions across the upper and lower limbs, MEPs in the wrist flexor and extensor are facilitated alternatively according to the flexion-extension phase of contralateral ankle movements^27^,^47^. The facilitation of hand-muscle excitability during simultaneous teeth clenching found in the present study might be due to similar muscle patterns in both motor acts (contraction, flexion). Future research should address whether extension and contraction of facial muscles alone or along with congruent movements in other limbs would result in a phase-dependent sinusoidal modulation of CSE in remote resting segments. Additionally, the effect of simultaneous foot dorsiflexion and teeth clenching on enhancement of force produced by arm or hand needs to be studied. It has already been shown that teeth clenching during MVC exertion showed larger maximal handgrip force^48^. Similarly, behavioral studies need to address how combined movements of foot and mouth could aid the coordination of hand movements. Musicians seem to take advantage of this mechanism by keeping a steady beat by foot tapping and humming the melody or rhythm while playing a musical instrument. Although this strategy is widely used among musicians, no research has yet empirically investigated it. Furthermore, this result might have an indirect implication for studies investigating the effect of language production on the excitability of M1. It has already been shown that the contraction of the muscles around oral organs such as the jaw muscles increases the excitability of the left M1 hand area, whether it is teeth clenching^3^ or producing vocal sounds^9^. The effect of articulation on hand motor excitability has been interpreted as an indication of the specific functional connection between the hand motor area and the cortical language network^49^. Hence, it remains unclear to what extent this facilitation could be related to language functions or the movement per se. One possible way to assess it would be to test for differences in hand-muscle MEP amplitudes during silent vs. overt articulation and during facial vs. foot movements.

Overall, our results imply that movement preparation and execution can lead to a widespread facilitation of excitability in M1, with combined movements imposing additional load. The higher CSE of a resting muscle induced by combined movements of other body parts may be relevant for post-stroke physical rehabilitation emphasizing isolated^50^ or bilateral movement training^51^. TMS studies have shown that cortical excitability in the affected motor cortex is reduced after a stroke^52^. Future research should address whether interventions designed to enhance the excitability of the paretic effector could be more efficient if they also involved the movement of body parts located remotely from the stroke-damaged area. Consider, for example, training the foot to rehabilitate a paralyzed hand rather than just using the affected or the homologous unaffected body parts such as merely a contralateral hand. This notion has not yet been studied adequately. It is noteworthy that in the present study participants performed ipsilateral foot dorsifelexion; admittedly, including contralateral dorsiflexion would be more sensible in terms of clinical applications, since arm dysfunction after stroke frequently occurs with dysfunction in the ipsilateral leg^53^. It has already been reported that unilateral muscle contraction leads to facilitation of MEP in the muscles of both ipsilateral and contralateral sides of distant segmental limbs^12^,^14^,^54^; however, the magnitude of facilitation has not been directly investigated across muscle pairs belonging to the same and the opposite side of the body—for example, left upper and left lower limb muscles versus left upper and right lower limb muscles. Hence, movements not only with ipsilateral but also with contralateral foot and even both feet would be relevant clinically.

## Materials and Methods

### Participants

A total of 17 healthy right-handed native Finnish speakers participated in the experiment (5 males and 12 females, age: 23.9 ± 4.2) after giving their written informed consent. They were rewarded for participating with movie tickets. The study was approved by the Ethical Review Board in Humanities and Social and Behavioural Sciences at the University of Helsinki and was carried out in agreement with legal requirements and international norms (Declaration of Helsinki, 1964).

### Experimental protocol

Participants were seated comfortably with their backs supported, heads placed into a headrest, elbows flexed to approximately 90° and forearms resting on the armrests of a chair. Their both feet were fully supported on a flat surface, and they were at the same height during the resting condition. Each session started with 3-second maximum voluntary contractions (MVC), performing three teeth clenchings and three right-foot dorsiflexions separately with a two-minute rest between each contraction. Participants were instructed to perform MVC at a level they felt comfortable even when maintaining the contractions over an extended period. They were told to perform either a right-foot dorsiflexion or teeth clenching when the word “Contract” appeared on the screen following a 3-second countdown, and to stop the contraction when the word “Relax” was shown. During foot dorsiflexion, the right heel remained on the surface while the ankle was fully dorsiflexed. The electromyographic (EMG) activity of the right masseter (MS) and the right tibialis anterior (TA) muscles were recorded simultaneously, and the average of root-mean-square (RMS) EMG amplitude over 3-second epochs for each muscle was used to define MVC.

The experiment consisted of 2 baselines and 3 experimental blocks, with a five-minute rest between the blocks. The first (*pre*) and the last (*post*) blocks were the baselines, each consisting of 15 trials, during which participants were instructed to keep their gaze on the fixation cross displayed in the center of the screen for 3 s. TMS pulses were delivered randomly within 700 ms after the presentation of the fixation cross at inter-stimulus intervals of 15 s, and MEPs were recorded from the right first dorsal interosseous muscle (FDI). Each of the three experimental blocks consisted of a set of 30 trials; TMS-evoked MEPs from the resting right FDI muscle were recorded during 3 tasks: (i) right-foot dorsiflexion (*FD*), (ii) teeth clenching (*TC*), and (iii) simultaneous right-foot dorsiflexion and teeth clenching (*FD & TC*) at 2 force levels: 0% and 100% of the MVC. The 3 tasks were randomly distributed over trials within each block. Each trial started with a fixation cue, presented for 1000 ms followed by a 50-ms visual stimulus that depicted the action to be performed. Participants were asked to perform an MVC of either teeth clenching or right-foot dorsiflexion or a combination of the two with a maximum effort and as fast as possible after the presentation of the visual stimulus. TMS pulses were delivered 180 ms after the presentation of the visual stimulus, when the muscles were still at rest (0% MVC). This delay allowed us to investigate the effects of movement preparation on evoked responses preceding the onset of voluntary EMG^55^. To obtain MEPs during 100% MVC, we used closed-loop real-time EMG signal analysis to deliver TMS pulses when RMS EMG amplitude in the tibialis anterior muscle (during *FD* task) or the masseter (during the *TC* task) or both of them (during *FD & TC* tasks) had maintained the value of MVC, which was the average RMS value of the three 3-second MVCs for a period of 500 ms (Fig. 1). This ensured that during all the 100% MVC conditions for each participant, the level of EMG activity remained unchanged when the TMS pulses were delivered. However, there were inter-individual differences in the range of activities, which is reflected in the results of EMG activity during the 100% MVC, reported in Supplementary Table S2. This implies that subjects with different degrees of strength were producing MVCs at the same relative value. The use of absolute test value for all the participants would cause subjects to produce MVCs at different relative levels, which would affect the CSE excitability in an uncontrolled manner.

The experiment was designed using Matlab 2014b (The Mathworks, Natick, MA), including the open-source Psychophysics Toolbox 3 (www.psychtoolbox.org) to control stimulus presentation and randomization of trials and to trigger TMS pulses.

### Transcranial magnetic stimulation (TMS)

A navigated transcranial magnetic stimulation (nTMS) system with a figure-of-eight coil was used to deliver the electromagnetic stimuli (*Nexstim Plc*, Helsinki, *Finland*). The coil was placed tangentially over the scalp with its handle pointing backward, and TMS was delivered to the optimal spot in the left M1; i.e., the location at which MEPs of maximal amplitude were induced in the contralateral FDI muscle. The intensity of stimulation over the left primary motor cortex was adjusted to 120–130% of the resting motor threshold to evoke MEPs with a peak-to-peak amplitude of about 1.0 mV. The resting motor threshold was defined as the minimum TMS intensity at which MEPs with peak-to-peak amplitudes of larger than 50 μV were induced in the FDI muscle in at least five out of ten successive trials under resting condition. By using the navigation system, the optimal stimulation spot was marked on the template MRI.

EMG was recorded from the FDI and the right tibialis anterior (TA) by using paired Ag/AgCl surface electrodes in a belly-tendon montage and from the right masseter (MS) by placing the electrodes 20 mm apart along the muscle-fiber direction. The EMG signals were band-pass-filtered online (10–500 Hz), amplified and sampled at the rate of 3 kHz.

### Data analysis

MEP peak-to-peak amplitudes from FDI were calculated off-line using Matlab 2014b. For 0% MVC, trials with pre-trigger background EMG on either TA or MS muscles were discarded from further analysis (3.4%) when the RMS EMG during the 50 ms before TMS pulse exceeded the RMS EMG at rest (during the 50 ms prior to the onset of visual stimulus) by more than two standard deviations. To reduce inter-subject variability, MEP amplitudes were transformed to their corresponding z-scores based on individual means and standard deviations across all the conditions. To validate the methodology of this study, following parameters are reported in Supplementary information: EMG activity during the movement preparation (0% MVC) and execution (100% MVC), TMS timing relative to EMG onset and MEP amplitudes in millivolt.

Normal distribution was tested by the Shapiro–Wilk test for each category of the independent variables. Moreover, the homogeneity of variances was assessed by Mauchly’s test of sphericity. Two one-way ANOVAs were used to examine the differences in MEP amplitudes elicited during different conditions. In all the ANOVAs, post-hoc comparisons were performed by means of t-tests, applying the Bonferroni correction. To estimate the effect sizes, we used Cohen’s *d* and partial eta-squared (η^2^) measures. Statistical significance was set to α = 0.05. Results are reported as mean ± standard error of the mean of MEP amplitudes in z-scores.

## Additional Information

**How to cite this article:** Komeilipoor, N. *et al*. Preparation and execution of teeth clenching and foot muscle contraction influence on corticospinal hand-muscle excitability. *Sci. Rep.* **7**, 41249; doi: 10.1038/srep41249 (2017).

**Publisher's note:** Springer Nature remains neutral with regard to jurisdictional claims in published maps and institutional affiliations.

## Supplementary Material

Supplementary Information
