# Supplementary material for: Preparation and execution of teeth clenching and foot muscle contraction influence on corticospinal hand-muscle excitability
Source: Sci Rep. 2017 Jan 24;7:41249. doi: 10.1038/srep41249 (PMC5259748; doi:10.1038/srep41249)
Supplement: Supplementary Information [file srep41249-s1.pdf]

Title: Preparation and execution of teeth clenching and foot muscle contraction influence corticospinal hand-muscle excitability

Authors: Naeem Komeilipoor*[a], Risto J. Ilmoniemi[b], Kaisa Tiippana[a], Martti Vainio[c], Mikko Tiainen[a], Lari Vainio[a]

Affiliations:

[a] Division of Cognitive and Neuropsychology, Institute of Behavioural Sciences, University of Helsinki, Finland

[b] Department of Neuroscience and Biomedical Engineering, Aalto University School of Science, Espoo, Finland

[c] Phonetics and Speech Synthesis Research Group, Institute of Behavioural Sciences, University of Helsinki, Finland

* **Corresponding Author**: naeem.komeilipoor@helsinki.fi

Supplementary Information

EMG activity during the movement preparation (0% MVC). To confirm that the resting state was maintained during movement preparation (0% MVC), we calculated the average root mean square (RMS) values of EMG signals during 50 ms before TMS pulse delivery in the right tibialis anterior (TA) and the right masseter (MS) muscles for each condition. The levels of RMS values during the rest were comparable in both muscles across different conditions. This confirms that the resting state was maintained during different conditions.

		**0% MVC**		
		FD	***TC***	***FD & TC***
**Muscles**	**TA**	**9.77 ± 0.99**	**9.82 ± 0.98**	**9.93 ± 0.90**
	**MS**	**9.32 ± 0.96**	**9.11 ± 0.94**	**9.13 ± 0.83**

"Supplementary Table S1: The grand mean (± SE) of RMS EMG values (μV) 50 ms before TMS in 0% MVC in the right tibialis anterior (TA) and the right masseter (MS) muscles during right-foot dorsiflexion (*FD*), teeth clenching (*TC*), and simultaneous right-foot dorsiflexion and teeth clenching (*FD & TC*) conditions."

EMG activity during the 100% MVC. To confirm that the contraction level was not different among conditions in each muscle, we computed the average RMS values of EMG signals 50 ms before TMS pulse delivery in TA and MS muscles for each condition. The levels of EMG activity during 100% MVC were comparable in each muscle across different conditions. Please note that the RMS of leg (TA) muscle during the teeth clenching (9.76) and that of face muscle (MS) during right foot dorsiflexion (9.48) were similar to the RMS levels at rest (See above table).

		**100% MVC**		
		FD	***TC***	***FD & TC***
**Muscles**	**TA**	**365.98 ± 19.82**	**9.76 ± 0.92**	**353.60 ± 30.01**
	**MS**	**9.48 ± 0.86**	**140.46 ± 12.21**	**136.42 ± 12.47**

"Supplementary Table S2: The grand mean (± SE) of RMS EMG values (μV) 50 ms before TMS in 100% MVC in the right masseter (MS) and the right tibialis anterior (TA) muscles during right-foot dorsiflexion (*FD*), teeth clenching (*TC*), and simultaneous right-foot dorsiflexion and teeth clenching (*FD & TC*) conditions."

TMS timing (ms) relative to EMG onset. Before designing the experiment, we were aware that delivering TMS pulses relative to presentation of visual cues or EMG onset would lead to stimulating the M1 while different muscles are in different levels of activity and that was the reason why we delivered TMS pulses based on the level of EMG activity. However, this choice resulted in different TMS timing relative to EMG onset of different muscles (see table below).

The EMG onset was defined based on a threshold method first described by Di Fabio [1]. The EMG signals were first full-wave rectified and then filtered using a low-pass filter with a cut-off frequency of 50 Hz. A window of 50 ms (i.e., prior to the presentation of visual stimulus) was used as the baseline. The onset was set at the first point when the change in filtered EMG signal exceeded 3 standard deviations above the mean baseline for 25 consecutive ms.

	**Tibialis Anterior (TA)**		**Masseter (MS)**	
	FD	***FD & TC***	***TC***	***FD & TC***
**0 % MVC**	**-212.67 ± 42.06**	**-235.06 ± 26.47**	**-198.60 ± 47.69**	**-243.22 ± 67.59**
**100 % MVC**	**775.69 ± 118.17**	**1009.87 ± 91.87**	**883.03 ± 127.58**	**977.90 ± 177.11**

Supplementary Table S3: The grand mean (± SE) of TMS timing (ms) relative to EMG onset of TA and MS muscles during 0% and 100% MVC of right-foot dorsiflexion (*FD*), teeth clenching (*TC*), and simultaneous right-foot dorsiflexion and teeth clenching (*FD & TC*) conditions.

MEP amplitudes (mV). To show that the transformation to z-score (reported in the manuscript) reduced intersubject variability, we calculated the average MEP amplitudes (mV) of FDI hand-muscle during different conditions and performed Shapiro–Wilk tests on MEP amplitudes in z-score and mV. The trend of excitability in raw MEPs and z-scores are similar. The Shapiro–Wilk tests revealed that MEP amplitudes (mV) in most categories of the independent variables were not normally distributed ($p < .05$) while were normalized for MEP amplitudes in z-score ($p > .05$). This shows that the transformation to z-score successfully reduced the variability.

	FD	***TC***	***FD & TC***	***Fixation***
**0 % MVC**	**3.53 ± 0.50**<br>***p = .014**	**3.34 ± 0.44**<br>***p = .010**	**3.78 ± 0.50**<br>***p = .006**	**2.50 ± 0.34**<br>***p = .026**
**100 % MVC**	**4.07 ± 0.55**<br>**p = .116**	**4.15 ± 0.51**<br>**p = .123**	**4.46 ± 0.53**<br>***p = .006**	

Supplementary Table S4: Grand mean (± SE) MEP amplitudes (mV) and p values tested by the Shapiro–Wilk test of FDI during observation of a fixation cross (Fixation), preparation (0% MVC) and execution (100% MVC) of right-foot dorsiflexion (*FD*), teeth clenching (*TC*), and simultaneous right-foot dorsiflexion and teeth clenching (*FD & TC*) conditions.

Reference

1 Di Fabio, R. P. Reliability of computerized surface electromyography for determining the onset of muscle activity. *Physical Therapy* **67**, 43-48 (1987).
